# Unraveling In-Situ Formation of Surface Nickel Nitride Structures in Plasma-Assisted Catalytic Ammonia Synthesis

**DOI:** 10.1021/acs.jpclett.5c03923

**Published:** 2026-02-17

**Authors:** Christopher Kondratowicz, Yiteng Zheng, Ning Liu, Ziqiao Chang, Yijie Xu, James L. Trettin, Bowen Mei, Bruce E. Koel, Yiguang Ju

**Affiliations:** † Department of Mechanical and Aerospace Engineering, 6740 Princeton University, Princeton, New Jersey 08544, United States; ‡ Department of Chemical and Biological Engineering, Princeton University, Princeton, New Jersey 08544, United States

## Abstract

We report the in situ formation of Ni nitride for plasma-assisted ammonia synthesis. Both the surface nitrogen concentration and the ammonia formation rate exhibit dependence on the N_2_:H_2_ feed ratio. The maximum surface nitrogen concentration occurs at a N_2_:H_2_ ratio of 4:1, and the maximum catalytic activity occurs at 2:1. In contrast, the formation of gas phase radicals is less sensitive to feed composition, indicating that Ni nitride is more kinetically relevant to ammonia production than gas-phase radicals. The plasma-induced formation of Ni nitride is therefore proposed to be a critical contributor to the synergistic effects in plasma-assisted catalytic ammonia synthesis. Additionally, Ni nitride alters the surface reaction mechanism of plasma-assisted ammonia synthesis, with the rate-determining-step (RDS) shifting to surface-bound NH_3_ formation rather than N_2_ activation at temperatures below 373 K. These findings provide mechanistic insight that opens opportunities for optimizing the performance of plasma-assisted catalytic ammonia synthesis.

## Graphical abstract

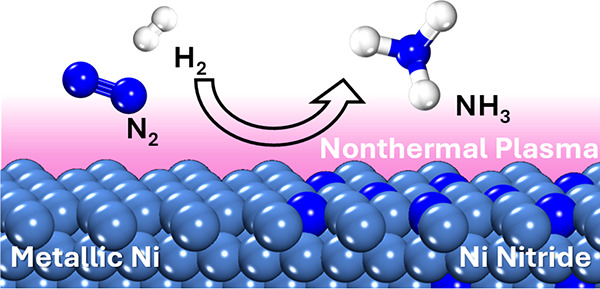

Ammonia is an essential chemical for fertilizer production and is being considered as a prospective carbon-free fuel.^1^ Most ammonia is produced via the Haber-Bosch process, which requires high temperature and pressure and is energy intensive with high CO_2_ emissions.^2^ Plasma-assisted catalytic ammonia synthesis is a promising alternative process to produce ammonia by using renewable electricity.^3–5^ Plasma-induced active species such as vibrationally and electronically excited molecules and radicals enable thermodynamically unfavored reaction pathways under typical thermal catalysis conditions.^6–12^ With these active species and novel reaction pathways, ammonia can be produced at milder reaction conditions.^3,4^ In addition, the presence of plasma can shift the ammonia synthesis volcano curve peak away from the laboratory benchmark Ru catalyst and toward non-noble catalysts such as Co and Ni.^5,9^

Although several aspects of plasma-assisted catalysis have been studied, including formation of N adatoms,^13^ gas-phase NNH formation,^7^ the effects of plasma-induced surface charge,^14^ and the modification of metal surfaces,^15,16^ a fundamental understanding of the changes produced by the synergy of the plasma and a heterogeneous catalyst is not yet clear. Additionally, a very recent study showed that plasma-induced WO_3_ nitridation could improve the catalytic activity for electrocatalytic ammonia synthesis.^17^ However, the dynamics of catalyst structures and active sites have not been systematically studied during plasma-assisted catalytic ammonia synthesis due to the challenges of in situ characterization of both the plasma and catalyst. Thus, the nature of catalytic active sites and reaction mechanisms for plasma-assisted catalytic ammonia synthesis are poorly understood.^18–22^ A better understanding of any dynamic transformation of the catalyst and its effect on the plasma properties is urgently needed to aid development of more energy efficient plasma-assisted catalytic processes for green ammonia synthesis. Herein, we report on studies of in situ surface nitridation of Ni catalysts and aspects of the catalytic reaction mechanism for plasma-assisted catalytic ammonia synthesis by combining in situ laser diagnostics, ex situ high-resolution X-ray photoelectron spectroscopy (HRXPS), and in situ diffuse reflectance infrared Fourier transform spectroscopy (DRIFTS).

Formation of nitride on a Ni foil catalyst exposed to plasma in an AC dielectric barrier discharge (DBD) reactor (schematic shown in Figure S1) using several N_2_/H_2_ ratios was examined using ex situ HRXPS (Figure 1). The AC DBD plasma was generated at 300 K, 100 Torr, 20 kHz, and 18 kV. The surface N concentration (mol %) obtained by HRXPS is shown in Figure 2B. Figure 1 also shows spectra from a Ni nitride reference sample that was obtained via thermal annealing in NH_3_.^23^ The two lowest binding energy (BE) peaks for Ni 2p_3/2_ (bottom spectrum in Figure 1A) were found at 852.6 and 853.8 eV, which are assigned to Ni^0^ from Ni metal and Ni^1+^ from Ni nitride (Ni_*x*_N_*y*_), respectively.^23,24^ Further oxidized species due to Ni^2+^ (854.4 eV) were observed,^25–28^ with corresponding satellite peaks at 856.1–860.9 eV. The Ni^2+^ peak can be further decomposed into more peaks that arise from multiplet splitting.^28–30^ We do not further investigate these complex multiplet structures since they are irrelevant to the formation of Ni nitride. N 1s spectra (Figure 1B) confirmed the formation of Ni nitride. The N 1s spectrum of the nitride reference sample showed a main peak at 397.9 eV, which is attributed to Ni nitride.^23,24,31^ Several minor peaks were also observed at 398.8–400.8 eV, which are assigned to adsorbed NH_*x*_ (x = 1, 2, and 3) species, since the Ni nitride reference sample was synthesized using NH_3_.^31,32^ For the Ni foil catalyst exposed to a pure-H_2_ plasma (top spectra in Figure 1), no N 1s peak was observed and only a Ni^0^ peak, along with oxidized Ni peaks due to transfer in air for ex situ analysis, was found in the Ni 2p_3/2_ spectrum. After pure-N_2_ plasma treatment of the Ni foil (next to the bottom curves in Figure 1), the Ni 2p_3/2_ and N 1s spectra very closely resembled the Ni nitride reference spectra. Minor NH_*x*_ peaks at 398.8–400.8 eV are also observed after N_2_ plasma treatment without H_2_ exposure. Two new peaks in Figure 1B were observed at 403.2 and 406.8 eV after N_2_-plasma treatment, which are assigned to Ni nitrate (Ni_*x*_(NO_3_)_*y*_).^33^ O 1s spectra were collected to further confirm the formation of Ni nitrate (). Three major O 1s peaks were observed for all the testing conditions herein: 530 eV for O of Ni oxide (NiO_*x*_), 531 eV for O of surface OH, and 533 eV for O of adsorbed H_2_O. An O 1s peak for Ni nitrate was observed at 536 eV only for pure-N_2_ plasma treated Ni foil, confirming the formation of Ni nitrate (Figure S2). The formation of oxidized Ni species and nitrates was likely due to a small presence of water during plasma exposures and/or sample transport in air for ex situ HRXPS analysis. The presence of these NH_*x*_ peaks is attributed to the interactions between Ni nitride and water adsorbed on the reactor walls prior to the experiments or during the sample transfer procedure. Ni nitride can react with water, particularly under plasma conditions, to form Ni­(OH)_*x*_ as well as adsorbed NH_*x*_ species.^34^

**Figure 1:**
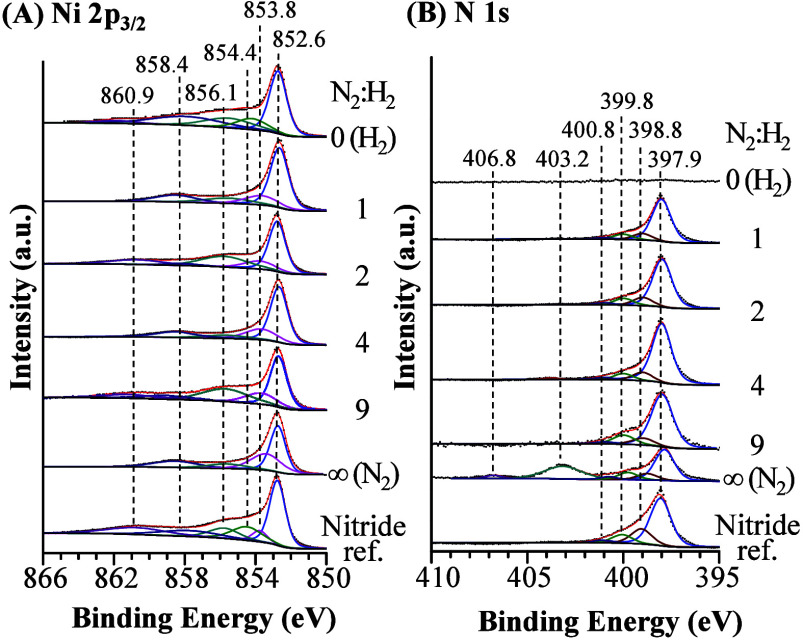
Ex situ HRXPS spectra for (A) Ni 2p_3/2_ and (B) N 1s regions from Ni foil treated by plasma using N_2_/H_2_, N_2_, and H_2_. The bottom spectrum in (A) and (B) is from Ni nitride reference material. AC DBD discharge conditions: 300 K, 100 Torr, 200 sccm, 20 kHz, and 18 kV.

**Figure 2:**
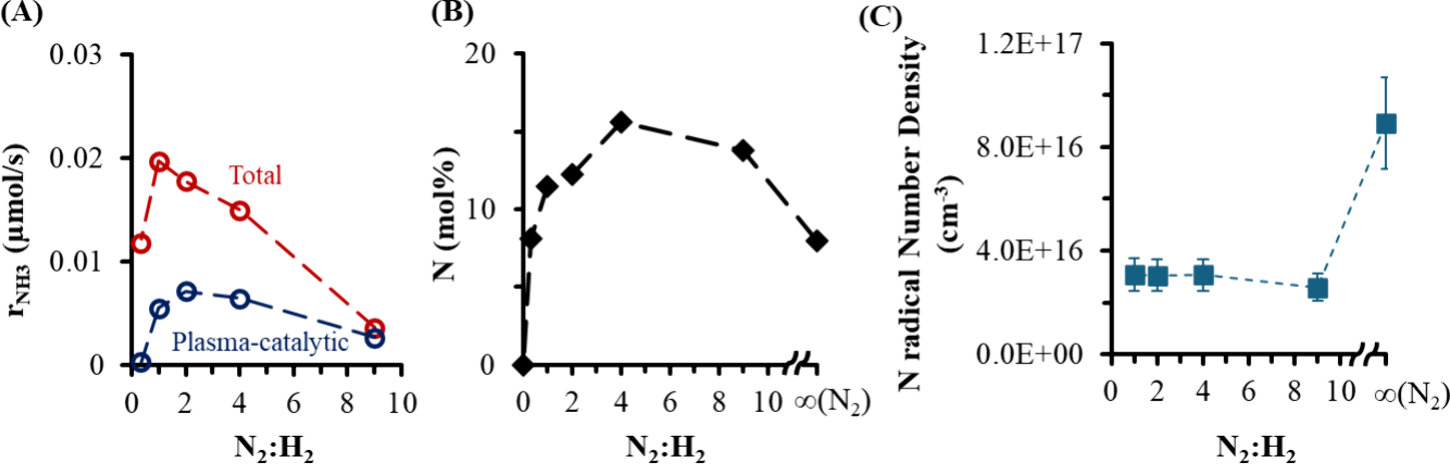
Effects of varying the gas-phase N_2_:H_2_ feed concentration on (A) total NH_3_ production rate (*r*_total_) and plasma-catalytic NH_3_ production rate (*r*_plasma‑catalytic_ = *r*_total_ – *r*_plasma‑only_), (B) N concentration at the surface (mol %) obtained by ex situ XPS, and (C) gas-phase N radical number density obtained by in situ TALIF. AC DBD discharge conditions: 300 K, 100 Torr, 200 sccm, 20 kHz, and 18 kV.

Figure 1 also shows that after exposure of the Ni foil catalyst to N_2_/H_2_-plasma for several different N_2_:H_2_ ratios, both the Ni 2p_3/2_ and N 1s spectra showed Ni nitride peaks, confirming the formation of Ni nitride under all the testing conditions in this study. The main Ni 2p_3/2_ peaks corresponded to Ni^0^ at 852.6 eV and Ni^1+^ of Ni nitride at 397.9 eV. A clear relationship between the surface N concentration and the N_2_:H_2_ ratio in the plasma was found (Figure 2b), with the largest surface N concentration at a N_2_:H_2_ ratio of 4:1. The addition of H_2_ in the plasma at relatively low concentrations (N_2_:H_2_ > 4:1) benefited the formation of Ni nitride, indicating a possible role of H-derived species (such as NH and H radicals) in surface nitride formation. Further increases in H_2_ at relatively higher concentrations (N_2_:H_2_ < 4:1) caused less surface nitride formation, which can be explained by two possible hypotheses. The first hypothesis is that surface N and H atoms can influence the activation barrier for the diffusion of surface N atoms into the subsurface. A high surface coverage of H atoms thus may reduce the nitridation rate. The second hypothesis is that plasma-induced etching of Ni nitride could occur at relatively high H_2_ concentrations in the plasma. Similar plasma-induced etching of the nitride has been proposed for an FeAl alloy treated with N_2_/H_2_ plasma.^35^

The ammonia formation rate in the plasma, r_NH3_, was the highest at an equimolar feed of N_2_:H_2_ = 1:1 (Figure 2A). A similar curve was reported for plasma-assisted ammonia synthesis over SiO_2_-supported Ni catalysts at 428 K, with the same optimal N_2_:H_2_ ratio.^36^ By subtracting the rate with and without the Ni catalyst, the synergy between the catalyst surface and the plasma, r_plasma‑catalytic_ (defined in the Supporting Information (SI), Section 1.3)^37^ for ammonia synthesis exhibited a strong correlation with the N_2_:H_2_ ratio, with the highest plasma-catalytic rate shifting to a larger N_2_:H_2_ ratio of 2:1 (Figure 2A). For plasma-assisted catalytic processes, the formation and loss rates of radicals and excited species in the plasma have been thought to play critical roles.^5,8,10,11^ Specifically, several studies concluded that N radicals were critical in producing ammonia.^8,10,11^ Therefore, in this study we focused on the presence of N and H radicals generated in the plasma. Measurement of the N and H radical number density was carried out with in situ two-photon absorption laser-induced fluorescence (TALIF) in the DBD reactor (Figure 2C and Figure S3). The maximum N radical number density was found in pure N_2_-plasma, and when H_2_ was added to form N_2_:H_2_ = 9:1, N radical number density decreased substantially. N radical number densities were nearly insensitive to further decreases in the N_2_:H_2_ ratio.^38^ In contrast to that for N radicals, the H radical number density was independent of the N_2_:H_2_ ratio in the study herein (Figure S3).

Ammonia formation rates did not align with the N and H radical number densities in the gas-phase plasma but were better correlated with surface N concentration (Figure 2). This indicates that ammonia formation was not controlled by the radical number densities alone, but rather the presence of surface nitride is indicated as kinetically relevant to ammonia formation rates. Metal nitrides have been reported to be active catalysts for ammonia synthesis without plasma,^39–41^ and N vacancies on nitride surfaces were proposed to be the most active sites for ammonia formation.^41,42^ Therefore, the high catalytic activity observed in the studies herein was attributed to the in situ formed surface Ni nitride. The discrepancy between the surface nitride concentration and ammonia formation rate is likely due to a lack of in situ information on the formation and concentration of N vacancies and active sites for ammonia synthesis.^41,42^ More sophisticated in situ and operando spectroscopy and imaging are needed to better understand the catalytically active sites.

To characterize surface-bound species on the catalyst during plasma-assisted catalytic ammonia synthesis, in situ DRIFTS experiments were conducted over prereduced Ni (metallic Ni) and pre-N_2_-plasma treated Ni (Ni nitride) catalysts. Density functional theory (DFT) calculations were performed to interpret the in situ DRIFTS results, and the same methodology has been employed in our previous studies.^43–46^ A DRIFTS reaction chamber equipped with a customized DBD plasma jet was used to acquire the in situ IR spectra under plasma-assisted reaction conditions, and the details of the plasma jet have been described in our previous study.^47^ The DBD plasma was generated at 10 Torr, 50 kHz, 3 kV, and 0.1 W. The plasma jet used for operando DRIFTS was operated under comparable conditions to the DBD reactor. Due to the experimental design, the plasma jet and DBD reactor cannot be operated under the same conditions, and the plasma power of the plasma jet (∼0.1 W) was lower than that of the DBD plasma reactor (∼3 W). Although the changes in plasma properties altered the relative concentrations of surface intermediates, the identities of these intermediates were not sensitive to the plasma power or the location of catalyst bed (within vs outside the plasma discharge).^19,48^ Therefore, we assume that similar activated species are formed in the plasma in these two setups, and that the reaction mechanisms are similar. Consequently, fundamental aspects of the reaction mechanism are transferable and useful.

The formation of Ni nitride after N_2_-plasma treatment was confirmed by Raman spectroscopy, temperature-programmed desorption (TPD) experiments, and high-angle annular dark-field scanning transmission microscopy/energy dispersive X-ray analysis (HAADF-STEM/EDX) (Figures S4, S5, and S7). On metallic Ni powder catalysts at 303 K (Figure 3A), IR bands at 1688 cm^–1^ and a broad peak centered at 3201 cm^–1^ are attributed to the asymmetric bending mode, δ_as_(NH_3_), and N–H stretching modes, ν­(N–H), respectively, of surface-bound NH_3_.^19^ We note that the broad peak at 3201 cm^–1^ is likely due to the heterogeneity of surface sites and the existence of different adsorbed NH_*x*_ species (Table S2). The peak at 1423 cm^–1^ is attributed to the asymmetric bending mode of surface-bound NH_2_ species, δ_as_(NH_2_).^19,49,50^ In addition, the peak at 1209 cm^–1^ is assigned to N-NH_2_ deformation, δ­(NNH_2_), of adsorbed NNH_2_ on the Ni surface.^19,49^ Gas-phase NNH_2_ radicals have been detected by molecular beam mass spectrometry during plasma-assisted ammonia synthesis, and similar adsorbed NNH_*x*_ species have been reported on different metal surfaces by transmission FTIR spectroscopy.^7,19^ The spectroscopic observation of NNH_2_ species on the surface strongly indicates that additional reaction pathways are enabled in the presence of plasma that do not occur during thermal catalysis. Other than Ni, adsorbed NNH_*x*_ species were found on surfaces of Fe, Co, and Ru.^7,19^ Therefore, formation of adsorbed NNH_2_ is expected to be important for plasma-assisted ammonia synthesis over a wide range of catalysts. Computational studies concluded that the formation of NNH_2_ could benefit ammonia synthesis,^51,52^ but there is no direct experimental data to prove the kinetic relevance of adsorbed NNH_2_ for plasma-assisted ammonia synthesis. Nevertheless, we suggest further consideration of the additional reaction pathways in which NNH_*x*_ species are involved, especially by use of microkinetic modeling and DFT calculations.

**Figure 3:**
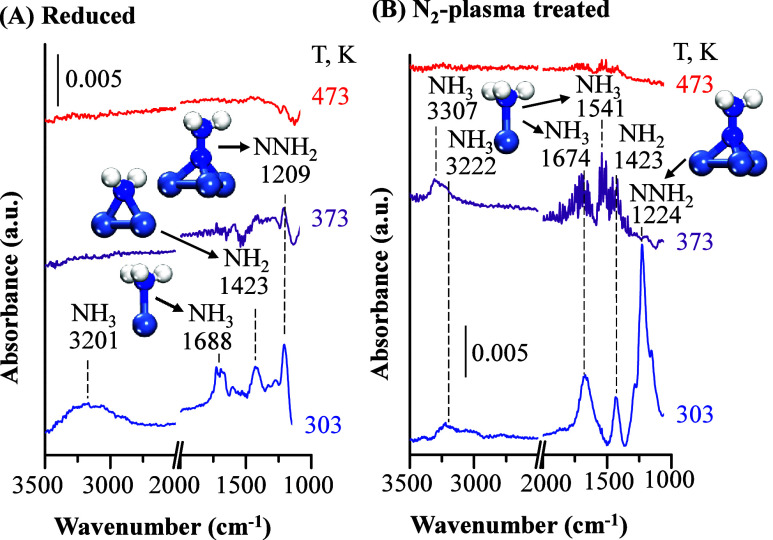
In situ DRIFTS spectra for (A) reduced (metallic Ni) and (B) N_2_-plasma treated (Ni nitride) Ni powder catalysts (unsupported) under plasma-assisted catalytic ammonia synthesis conditions. Spectra were obtained in a DRIFTS reaction chamber equipped with a customized DBD plasma jet. Discharge conditions: 300 K, 10 Torr, 100 sccm, N_2_:H_2_ = 4:1, 50 kHz, 3 kV, and 0.1 W. In the schematic drawings of the intermediates shown, atoms colored as Ni (blue-gray), N (blue), H (white).

Furthermore, Figure 3A shows that surface-bonded NH_2_ and NH_3_ species disappeared as the reaction temperature increased to 373 and 473 K. The exception was the peak for surface-bound NNH_2_, which was still detected at 373 K. The negative peaks observed at 373 K indicate more complex reactions may occur and further experiments are needed. The peak for surface-bound NNH_2_ was not detected at 473 K.

For N_2_-plasma pretreated Ni catalysts, similar peaks for surface-bound NH_2_, NH_3_, and NNH_2_ species were observed at 303 K, but the relative IR band intensities were changed (Figure 3B). The peak of adsorbed NNH_2_ at 1224 cm^–1^ was the most prominent, indicating the surface coverage of NNH_2_ species on Ni nitride was significantly higher than that on metallic Ni. The change in the surface coverage of NNH_2_ is due to the more extensive formation of Ni nitride on the N_2_-plasma pretreated catalyst, which favors an alternative reaction pathway by forming more adsorbed NNH_2_. At 373 K, the peak of NNH_2_ at 1224 cm^–1^ disappeared and the adsorbed NH_3_ peaks at 1674 and 1541 cm^–1^ and the adsorbed NH_2_ peaks at 1423 cm^–1^ were clearly smaller, but their analysis was obscured by the rotational–vibrational bands of gas phase ammonia. These bands from 1750 to 1250 cm^–1^ indicate more gas-phase ammonia was produced by the pretreated Ni nitride catalyst. When the reactor temperature was increased to 473 K, no surface-bound species were found. The IR peak assignments were confirmed by density functional theory (DFT) calculations, and the results are summarized in Tables S2 and S3.

The discovery of surface-bonded NH_2_ intermediate indicates that the reaction mechanism for catalytic ammonia synthesis could be tuned by Ni nitride formation under appropriate plasma discharge conditions. In a Langmuir–Hinshelwood (L-H) mechanism for thermal catalytic ammonia synthesis, the rate-determining-step is usually N_2_ activation (N_2_ = 2 N*).^2,53^ In plasma-assisted catalytic ammonia synthesis, with the increase of H atoms in plasma and lower energy barrier of N_2_ dissociative adsorption due to nickel nitride formation,^41,42^ an Eley–Rideal (E-R) mechanism can be dominant, with the rate-determining-step of surface-bound NH formation (N* + H = NH*, or N + H* = NH*).^6,8,12^ In both these reactions, the surface coverages of NH and NH_2_ adsorbed species should be very low, since they would be consumed immediately once formed.^8,54^ Due to the low surface coverage, no surface-bonded NH_2_ species should be detected on the catalytic surface if the rate-determining-step of plasma-assisted catalytic ammonia synthesis remained N_2_ activation or surface NH formation. It was proved by earlier observations that NH_3_ was the only detectable adsorbate in IRAS experiments for thermal catalytic ammonia synthesis.^55^

However, surface-bonded NH_2_ intermediate species were spectroscopically observed by in situ DRIFTS (Figure 3), which indicates that the rate-determining-step in plasma-assisted catalytic ammonia synthesis may switch to the formation of adsorbed NH_3_ (NH_2_* + H* (or H) = NH_3_*) or NH_3_ desorption at temperatures lower than 373 K. A simplified schematic for the proposed reaction mechanism is shown in Figure 4. A similar mechanism has been reported for thermal catalytic ammonia synthesis over Fe catalysts by using operando XPS and microkinetic modeling.^56^ The rate-determining step was proposed to be the formation of adsorbed NH_*x*_ over Fe(110) and Fe(210) surfaces at temperatures lower than 523 K. Therefore, by directly observing adsorbed NH_2_, the rate-determining-step for plasma-assisted catalytic ammonia synthesis can be suggested to switch to NH_3_ formation or desorption.

**Figure 4:**
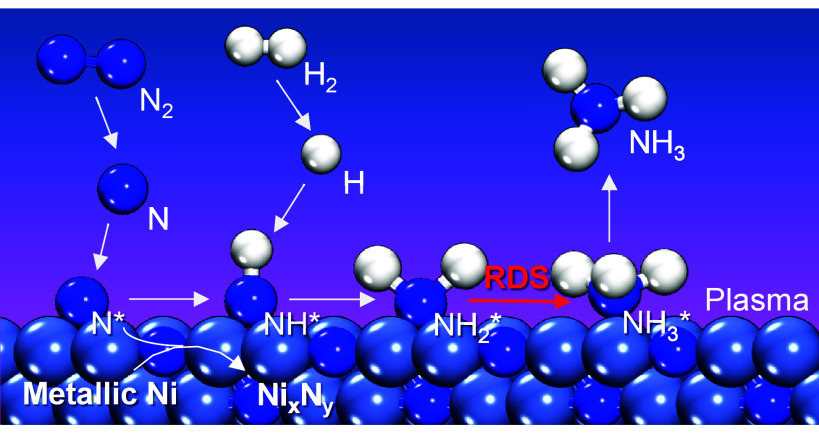
Schematic of proposed reaction mechanism for plasma-assisted catalytic ammonia synthesis on the in situ-formed Ni nitride in an N_2_/H_2_ AC-DBD plasma.

Additionally, the rate-determining-step for plasma-assisted catalytic ammonia synthesis was found to be sensitive to the reaction temperature. While the formation of adsorbed NH_3_ appears to be the rate-determining step at relatively low temperatures (≤373 K), at 473 K, the rate-determining-step switches back to N_2_ activation or N–H formation since no adsorbed NH_2_ was observed. The change of the rate-determining-step at low reaction temperatures was also observed on Fe catalysts for thermal catalytic ammonia synthesis.^56^ The in situ DRIFTS results herein suggest that the reaction mechanism of plasma-assisted catalytic ammonia synthesis on Ni catalysts can be tuned by varying the reaction temperature. Hydrogenation steps affected the overall ammonia synthesis rates on Ni catalyst surfaces at low reaction temperatures. These insights for plasma-assisted ammonia synthesis indicate that it is preferable to optimize reaction conditions and plasma properties to favor the hydrogenation reactions. We note that the active sites of ammonia synthesis have been proposed to be N vacancies of metal nitrides.^41,42,57,58^ However, it is challenging to experimentally observe surface N vacancies via the ex situ characterizations. The role of formation of N vacancies or the formation of gas phase radicals was not determined in the study.

The in situ DRIFTS results are consistent with our findings from XPS, reaction kinetics testing and in situ TALIF measurements. At relatively low H_2_ feed concentrations (N_2_:H_2_ > 4:1), the addition of H_2_ promoted the formation of Ni nitride (Figure 1), which has been proposed to be the active site for ammonia synthesis by lowering the activation energy of N_2_ activation.^59^ The presence of H_2_ also promoted the rate-determining-step and increased the formation of adsorbed NH_3_ as indicated by in situ DRIFTS. Consequently, the addition of H_2_ enhanced the NH_3_ production at N_2_:H_2_ ratios above 4:1 (Figure 2A). Furthermore, the introduction of H_2_ suppressed the formation of gas phase N radicals (Figure 2C), which has been identified as a key contributor to poor energy efficiency of plasma-assisted ammonia synthesis.^8^ Therefore, at low H_2_ concentrations, the present of H_2_ benefits the formation of NH_3_ for plasma-assisted ammonia synthesis.

However, at high H_2_ concentrations (N_2_:H_2_ < 4:1), competitive adsorption between surface N and H became dominant. Although H adsorption on Ni surface was weaker than that of N,^60^ the dissociation of H_2_ was significantly easier than that of N_2_. Consequently, a high surface coverage of atomic H inhibited the adsorption of N-related species and also suppressed the formation of catalytically active Ni nitride (Figure 1),^56^ resulting in a decreased NH_3_ synthesis rate at N_2_:H_2_ ratios below 4:1 (Figure 2A). Additionally, our previous study showed that excess H_2_ also favored the formation of gas phase NH radical via the V–V′ vibrational energy exchange, H_2_ (v)-N_2_ (v),^11^ which is energetically unfavorable.^8^ Overall, our results suggested that maintaining a relatively low H_2_ concentration (N_2_:H_2_ > 4:1) favored the formation of surface Ni nitride and enhanced the catalytic ammonia production under plasma-assisted conditions.

In summary, during plasma-assisted catalytic ammonia synthesis using a Ni catalyst, we identified the in situ formation of Ni nitride for the first time, which is suggested to contribute to high catalytic activity. Surface N vacancies are proposed to serve as the catalytic active sites for ammonia synthesis on various metal nitrides.^41,42,57,58^ The presence of N vacancies could lower the activation barrier of N_2_ dissociation. However, these surface N vacancies are not detectable by ex situ characterizations due to their high catalytic reactivity.^41,42,61^ To better understand the formation of N vacancies, we are continuing to develop in situ characterization tools under plasma-assisted reaction conditions. Furthermore, surface-bonded NH_2_ intermediates were identified using in situ DRIFTS experiments and we conclude that this species is a critical intermediate for plasma-assisted NH_3_ formation, which indicates a change of the thermal catalysis reaction mechanism for plasma-assisted catalysis at temperatures lower than 373 K. The reaction pathways of plasma-assisted ammonia synthesis appear to be altered and accelerated by the formation of Ni nitride, and the rate-determining-step of plasma-assisted ammonia synthesis can be shifted to surface-bound NH_2_ reactions to form adsorbed or gas-phase NH_3_ (NH_2_* + H* (or H) = NH_3_*). At high reaction temperatures (473 K), the rate-determining-step changes back to N_2_ activation or surface NH formation. In addition, surface-bound NNH_2_ species were found during plasma-assisted ammonia synthesis, but their kinetic relevance remains unclear.

The synergy between plasma and heterogeneous catalysis for ammonia synthesis can be attributed to two major factors. First, plasma-induced active species (N and H radicals in this study) facilitated additional reaction pathways in both the gas phase and on the catalyst.^5,8,11^ Second, reactive species generated in the N_2_/H_2_ plasma discharge enhanced surface nitride formation, thereby modifying the reaction mechanism of NH_3_ synthesis and altering the apparent activation energy of ammonia synthesis.^38,39,62,63^ The obtained fundamental understandings of the in situ formation of Ni nitride and its influence on the NH_3_ synthesis mechanism opens new opportunities for optimizing the catalyst for plasma-assisted catalytic ammonia synthesis. Beyond ammonia synthesis with Ni catalysts, these studies clearly show that it is important to consider the possible plasma-induced changes and reconstructions of the catalytic surface for a wide range of catalysts and plasma-assisted catalytic processes.

## Supplementary Material





## Notes

Detailed experimental and computational methods for catalysts pretreatments, in situ and ex situ characterizations and laser diagnostics (TALIF, XPS, DRIFTS, TPD, Raman, TEM, and XRD), and DFT calculations, supplementary discussion for the formation of Ni nitride over Ni powder, and supplementary figures and tables (PDF)
Transparent Peer Review report available (PDF)
