# Supplementary material for: Unraveling In-Situ Formation of Surface Nickel Nitride Structures in Plasma-Assisted Catalytic Ammonia Synthesis
Source: J Phys Chem Lett. 2026 Feb 17;17(13):3933–9. doi: 10.1021/acs.jpclett.5c03923 (PMC13051430; doi:10.1021/acs.jpclett.5c03923)
Supplement: Supplementary file 1 [file jz5c03923_si_001.pdf]

Unraveling In-situ Formation of Surface Nickel Nitride Structures in Plasma-Assisted Catalytic Ammonia Synthesis

Christopher Kondratowicz[1#], Yiteng Zheng[2#], Ning Liu[1], Ziqiao Chang[1], Yijie Xu[1], James L. Trettin[2], Bowen Mei[1], Bruce E. Koel[2*], Yiguang Ju[1*]

[1]Department of Mechanical and Aerospace Engineering, Princeton University, Princeton, New Jersey 08544

[2]Department of Chemical and Biological Engineering, Princeton University, Princeton, New Jersey 08544

[#] C.K. and Y.Z. contributed equally to this paper.

Corresponding authors: bkoel@princeton.edu, yju@princeton.edu

1. Experimental Methods

1.1 Ni Foil Catalysts Pretreatment

Ni foil (29×19×0.5 mm, Millipore Sigma, 99.98%) was used as a catalyst for in-situ/operando TALIF and ex-situ XPS analysis. Prior to the plasma treatment, the Ni foil was polished using an Allied Multi Prep Polisher. The Ni foil was first polished using 600-grit super fine sandpaper and then further polished using a chemical-mechanical polish to achieve a smooth Ni surface. The polishing slurry consisted of 60-wt% colloidal silica (average particle size of 7 nm, a 30-wt% suspension in H_2O, Sigma Aldrich, LUDOX SM) and 40-wt% H_2O_2 (Fisher Chemical, 30-wt%, certified ACS grade), and the slurry was adjusted to a pH of 5 by adding acetic acid (Sigma Aldrich, ACS reagent, 99.7%). After polishing, the Ni foil was rinsed with DI water and sonicated at room temperature for 30 min. The cleanness of Ni foil was checked by XPS before further experiments.

1.2 In-situ Two-Photon Laser Induced Fluorescence (TALIF)

In-situ TALIF measurements of N-radical and H-radical formation, as well as reaction rate measurements for ammonia production, were conducted using a quartz DBD plasma reactor with a 7-mm discharge gap (Figure S1). Flow rates of gas-phase reactants, N_2 and H_2, were controlled

using pre-calibrated mass flow controllers. The reactor temperature was kept at 300 K and the total pressure was maintained at 100 Torr.

A femtosecond (fs) laser system (Coherent Astrella amplifier and TOPAS OPA) generated UV fs pulses with a pulse energy of 3.5 μJ at a central wavelength of 205.08 nm for H radical measurements and a pulse energy of 2.6 μJ at a wavelength of 206.65 nm for N radical measurements at a repetition rate of 1 kHz. The laser beam has a diameter of 4 mm before being focused by a 250-mm focal length lens and sent into the reactor. The laser was parked in the middle of DBD plasma reactor (about 3 mm above the catalyst, Figure S1) to excite the 1s $^2S_{1/2} \rightarrow$ 3d $^2D_{3/2,5/2}$ transition of the H radical or the $2p^3$ $^4S_{3/2} \rightarrow$ 3p $^4S_{3/2}$ transition of the N radical. Using Gaussian beam optics and a center wavelength of 206.65 nm (for N atoms), the beam diameter at the focal point was 16.4 μm. The H fluorescence signals emitted at 656.3 nm and the N fluorescence signals emitted at 742–746 nm from the plasma were separately imaged by an intensified charge coupled device (ICCD) camera (Princeton Instruments PIMAX-4). For H fluorescence, a bandpass filter with a full-width-at-half-maximum (FWHM) of 10 nm centered at 656 nm was placed in front of the camera lens to block any scattered light from the laser and plasma emissions. For N fluorescence signals, a bandpass filter with a 10-nm FWHM centered at 745 nm was used. The camera was synchronized with the laser using control electronics, and the gate time was 110 ns for N and 200 ns for H. 5000 shots were averaged for each measurement to obtain a good signal-to-noise ratio. To obtain the absolute concentrations of H and N, the TALIF calibration for the N and H number density was conducted using the krypton method as reported previously, because N, H, and Kr share similar excitation and fluorescence wavelengths.[S1] The two-photon absorption cross-section ratio of Kr/N and Kr/H were 0.67 and 0.027, respectively.[S2-S3]

1.3 Reaction Testing

Reaction testing for plasma-assisted ammonia synthesis over a Ni foil catalyst was conducted using a quartz DBD plasma reactor designed and made in-house for in-situ TALIF, with four-way optical access (plasma volume of 6.9 cm^3). A schematic of the reactor is shown in Figure S1. The polished Ni foil catalyst was treated in this reactor, first by reduction in a H_2-plasma with a 100-sccm flow of H_2 (Airgas, UHP5.0, 99.999%) at 300 K and 100 Torr for 1 h. After reduction, the feed gas was switched to a 200-sccm flow of N_2 (Airgas, UHP5.0, 99.999%) or N_2/H_2 at 300 K and 100 Torr. DBD plasma was generated by an AC power source (Information Unlimited, PVM500) using a frequency of 20 kHz and an applied voltage of 18 kV for all plasma discharges. The applied voltage was recorded by a high-voltage probe (Tektronix, P6015A) and an oscilloscope (Tektronix, TDS2012B).

Reaction products were analyzed after 60 min of plasma treatment using an online-sampling Agilent 8890 gas chromatograph (GC) equipped with a nitrogen-chemiluminescence detector (NCD) and a CP-volamine column (CP7448, 60 m, 0.32 mm, Agilent). A two-stage gas-sampling system was developed to pressurize the outlet gas to 1 atm for consistent GC sampling

without interfering with the pressure and the plasma properties in the reactor. The sampling system was used and described in our previous studies.[S4-S5] Reaction conditions were chosen to limit the N_2 conversion to be less than 1% so that a differential reactor model could be used. The catalytic activity of the Ni foil catalyst in Figure 2a was reported as the total reaction rate of ammonia formation, r_{NH3}. The total ammonia formation reaction rate was calculated using the following equation:[S6]

$$r_{NH3}(\frac{\mu mol_{NH3}}{s}) = [p_{NH3}^{out}(ppm) - p_{NH3}^{in}(ppm)] \cdot \dot{V}_{total}(sccm) \cdot 7.45 \times 10^{-7}(\frac{\frac{mol}{s}}{sccm}) \cdot\ 10^{6}(\frac{\mu mol}{mol})$$

where p_{NH3} is the partial pressure of NH_3 that was directly measured using an online-sampling GC, and $\dot{V}_{total}$ is the volumetric flow rate of the gas feed. Reaction rates reported were obtained from the average of three independent runs at steady state. The reaction rates and the corresponding N_2 conversions are summarized in Table S1.

To deconvolute the contribution of the gas-phase plasma reactions and surface catalytic reactions, we assumed that the apparent ammonia formation rates were the summation of the following rates:[S7]

$$r_{NH3} = r_{plasma-only} + r_{thermal-only} + r_{plasma-catalytic}$$

where $r_{plasma\text{-}only}$ is the ammonia formation rate via homogeneous plasma-phase reaction, $r_{thermal\text{-}only}$ is the ammonia formation rate via the catalytic surface in the absence of plasma-derived activated species, and $r_{plasma\text{-}catalytic}$ is the ammonia formation rate via the synergies between the catalytic surface and plasma. We note that the actual reaction mechanism and ammonia formation pathways are more complex than given by this simplified equation.[S1, S8-S12] In this study, we only use this simplified rate expression to qualitatively examine the contribution of plasma-assisted catalytic reactions with different N_2:H_2 gas feed ratios.

No ammonia was detected using the Ni catalyst without plasma in the DBD reactor at all the N_2:H_2 concentrations studied herein. So, the Ni catalyst did not catalyze ammonia synthesis without plasma under the testing conditions herein (300 K, 100 Torr). Therefore, the $r_{thermal\text{-}only}$ is negligible in these studies, and the equation above can be further simplified to:

$$r_{NH3} = r_{plasma-only} + r_{plasma-catalytic}$$

With this equation, we can obtain the values of $r_{plasma\text{-}catalytic}$ by subtracting $r_{plasma\text{-}only}$ from r_{NH3}. The $r_{plasma\text{-}only}$ values were obtained using the same reactor and under identical plasma reaction conditions without adding the Ni foil. Since we only added a small Ni foil in the flow reactor, we assumed that the plasma was not significantly affected by the addition of Ni. The $r_{plasma\text{-}only}$ was assumed to be independent of the existence of the Ni catalyst.[S7-S8]

1.4 Ex-situ High-Resolution X-Ray Photoelectron Spectroscopy (HRXPS)

HRXPS analysis of the plasma-pretreated Ni foil catalyst was conducted using a ThermoFisher K-Alpha X-ray photoelectron spectrometer equipped with a monochromatic X-ray source and a focusing lens allowing for analysis areas from 30 to 400 μm. No charge neutralizer was used since the Ni foil samples were conductive. Broad scan survey spectra were taken at 200 eV pass energy and a 10 ms dwell time, and the specific region spectra (Ni 2p, O 1s, and N 1s) were collected at 50 eV pass energy and a 50 ms dwell time. The analysis spot size was 400 μm for all measurements. XPS binding energies are reported referenced to adventitious carbon with a C 1s peak at 284.0 eV.

The Ni nitride reference sample used for these XPS measurements was synthesized following a procedure reported previously.[S13] Since our DBD plasma reactor cannot be heated to the desired temperature, a quartz tube reactor was used to synthesize Ni nitride, with the details of this reactor setup as described previously.[S14] A polished Ni foil was reduced in a 50-sccm flow of 20 mol% H_2/Ar at 723 K for 1 h in the flow reactor. The reduced Ni foil was then treated thermally in a 50-sccm flow of 3 mol% NH_3/N_2 (Airgas, certified standard) at 623 K for 4 h to form Ni nitride. The Ni nitride reference sample was then cooled to room temperature under the same NH_3/N_2 flow.

1.5 Ni Powder Catalyst Pretreatment

The Ni foil catalyst was not amenable for characterization using available setups for Raman, TPD, XRD, TEM, and DRIFTS. Therefore, complementary in-situ and ex-situ characterization experiments were conducted using unsupported Ni powder catalysts. Although the physical properties of Ni foil and Ni powder are different, we assume that the surface chemistry and reaction mechanisms for these two types of Ni catalysts are similar.

Ni powder (Sigma Aldrich, <50 μm, 99.7% trace metal basis) used as a catalyst was characterized by Raman, TPD, XRD, TEM, and in-situ DRIFTS. The Ni powder was sieved using a 635 mesh to make sure all the Ni particles were less than 20 μm in these studies. Prior to characterization, a Ni powder catalyst was thermally reduced in a quartz coaxial DBD plasma reactor that was described previously.[S9] The catalyst was dried in a 50-sccm flow of Ar (Airgas, UHP5.0, 99.999%) by ramping the temperature from 303 K to 383 K with a ramp rate of 5 K/min and holding at 383 K for 1 h. After drying, the catalyst was reduced in a 50-sccm flow of 20 mol% H_2/Ar by increasing the temperature to 723 K with a ramp rate of 10 K/min and holding for 1 h. After reduction, the Ni catalyst was cooled to 303 K in a 50-sccm flow of Ar, with the pressure of the system adjusted to 100 Torr by using an in-line oil-free vacuum pump (Anest Iwata Corporation, ISO-250B) and a needle valve. When the temperature and pressure had stabilized, the gas feed was switched to a 50-sccm flow of 40-mol% N_2/Ar. N_2-plasma was generated by an AC power source with a frequency of 20 kHz. The applied voltage and the voltage of an external capacitor were recorded by an oscilloscope. The catalyst was treated in this N_2-plasma at 303 K for 1 h. After this treatment, the catalyst was kept under vacuum no longer than 48 h before characterization. The plasma conditions were selected such that the Ni foil and powder catalysts

were treated under comparable plasma conditions. The formation of Ni nitride over Ni powder catalysts was confirmed by ex-situ HRXPS.

1.6 Raman Spectroscopy

Ex-situ Raman spectra of Ni powder catalysts were obtained with a high-resolution, dispersive Raman spectrometer system (Horiba LabRAM ARAMIS) equipped with four laser excitations (325, 532, 633, 785 nm). We used the 532 nm (green) laser in this study. The Ni powder catalyst was transferred in a glove box filled with Ar from the coaxial reactor to a home-made Raman cell with a quartz window and O-ring seals. The Raman cell was evacuated and then sealed with a pressure of 10 mTorr. Raman spectra were collected using the Raman cell under vacuum at 303 K. For comparison, the Raman spectrum of the reduced Ni powder without plasma exposure was also collected using the same Raman cell.

The laser was focused on the sample with the confocal microscope equipped with an Olympus MPlan N 50x objective. The Raman spectrometer was optimized for the best spectral resolution by employing a 1200 grating and a 200-µm aperture. A ND 0.3 filter was used. The wavenumber calibration of the Raman spectrograph was checked using the silicon line at 520.7 cm^{-1}. The Raman spectra were collected by averaging 5 scans at 30 s/scan.

1.7 Temperature Programmed Desorption (TPD)

TPD experiments were conducted using a coaxial tube reactor.[S9] The Ni powder catalyst was first treated in-situ using an N_2 plasma following the procedure given in Section S1.5. Then the catalyst was purged under a 50-sccm flow of Ar, and the pressure was allowed to stabilize at 1 atm. After the pressure stabilized, the temperature was ramped from 300 K to 800 K at a ramp rate of 10 K/min. For comparison, a Ni powder catalyst was treated following the same pretreatment procedure and using the same reactor, but without a plasma discharge. An online-sampling mass spectrometer (MS, SRS, RGA 100) was used to analyze the gas outlet of the reactor by monitoring the following mass/charge (m/z) ratios: 2 (H_2), 12 (C), 14 (N), 18 (H_2O), and 28 (N_2).

1.8 X-Ray Diffraction (XRD)

Ex-situ XRD patterns of the N_2-plasma treated Ni powder catalysts and reduced Ni powders were collected using a Bruker D8 Discover Diffractometer with Cu Kα radiation at a wavelength (λ) of 0.15406 nm and operated at 40 kV and 40 mA. The catalyst samples were pressed and formed into a thin wafer on microscope slides (Superfrost Plus, Fisher Scientific). The spectra were collected over a 2θ range of 10-80° with a step size of 2°/min. The XRD reference spectra of bulk metallic Ni and Ni_3N were generated in Materials Studio 2019 software by Dassault Systèmes BIOVIA,[S15] and the crystallographic information files (CIF) were obtained from the Materials Project database (mp-23 for Ni and mp-2033 for Ni_3N).

1.9 High-Angle Annular Dark-Field Scanning Transmission Microscopy/Energy Dispersive X-ray Analysis (HAADF-STEM/EDX)

Ex-situ microscopy measurements were performed with a FEI Talos F200X microscope using a spherical aberration corrector on the probe-forming lens at an accelerating voltage of 200 kV. The Ni powder after N_2-plasma treatment was ultrasonicated in acetone (Sigma Aldrich, ACS reagent, >99.5%). Then, a few drops of the produced suspension were placed on a carbon-coated copper grid (Electron Microscopy Science, LC200-Cu-25). Afterward, the sample was allowed to dry at room temperature in a vacuum oven. Energy dispersive X-ray spectroscopy (EDS) mapping was conducted using ChemiSTEM quad detectors at a current of 0.12 nA for 15 min.

1.10 In-Situ Diffuse Reflectance Infrared Fourier Transform Spectroscopy (DRIFTS)

In-situ DRIFTS spectra under N_2/H_2-plasma conditions were collected using a Bruker INVENIO FTIR spectrometer equipped with a liquid nitrogen cooled mercury-cadmium-telluride (MCT) detector, a Harrick Praying Mantis diffuse reflection accessory, and a Harrick Praying Mantis high temperature reaction chamber equipped with two ZnSe windows. A plasma jet was designed and constructed in-house using polyetheretherketone (PEEK) for coupling with the DRIFTS dome and exposing the afterglow of the DBD plasma to the catalyst bed. Details of the plasma jet and DRIFTS reaction chamber were described in our previous work.[S16]

About 0.02 g of the Ni powder catalyst was pretreated in a coaxial quartz reaction following the procedure given in section S1.5. The Ni catalyst samples were transferred from a quartz reactor to the in-situ DRIFTS reactor inside a glove box filled with Ar. Then the catalyst samples were dried in a 50-sccm flow of Ar at 373 K for 30 min. After drying, the temperature was then stabilized at 303 K, 373 K, and 473 K under a 50-sccm of Ar flow while the pressure was allowed to decrease to 10 Torr. After the pressure and temperature stabilized, the gas feed was changed to a 50-sccm of 40 mol% N_2/10 mol% H_2/Ar. The N_2/H_2-plasma was generated by an AC power source with the applied voltage set to 3 kV and a plasma power of 0.1 W. After the plasma was turned on and stabilized for 5 min, the spectra were collected at a resolution of 4 cm^{-1} and averaging 100 scans. Baselines were collected for Ni catalyst samples at each temperature under N_2/H_2 flow without turning on the plasma discharge.

1.11 Dispersion-Corrected Density Functional Theory (DFT-D) Calculation

Gradient-corrected spin-polarized periodic DFT calculations with the $DMol^3$ code in Materials Studio 2019 by Dassault Systèmes BIOVIA Corporation were performed for determining geometries, adsorption energies, and the corresponding vibrational frequencies of possible intermediates of ammonia synthesis (NH_x and NNH_x) on infinite Ni(111) and Ni_3N(001) surfaces constructed by using periodic unit cells.[S13] The Ni(111) unit cell was generated from the bulk crystal using the optimized lattice constant of 0.3597 nm for Pt (within 2% of the 0.3524 nm experimental value) and contained 9 surface Ni atoms with 4 layers for a total of 36 Ni atoms. The surface coverage was 1/9 ML: one NH_x or NNH_x on the surface with 9 Ni atoms. The Ni_3N(001) unit cell was generated from the CIF file obtained from the Materials Project database (mp-2033) using the optimized lattice constant: a = b = 0.4433 nm, c = 0. 4411 nm. The Ni_3N(001) unit cell contained 12 surface Ni atoms and 4 surface N atoms with 4 layers for a total 48 Ni atoms and 16

N atoms. The surface coverage was 1/12 ML: one NH_x or NNH_x on the surface with 12 Ni atoms. For both Ni(111) and Ni_3N(001), a vacuum spacing of 4 nm in the c direction was used, and the top two layers of the surfaces were optimized with adsorbates during the geometry optimization, simulating surface relaxation after adsorption. The remaining two bottom layers were constrained at the bulk crystal positions, simulating the bulk structure.

The calculations used the double numerical with polarization (DNP) basis set and the generalized gradient-corrected Perdew-Burke-Ernzerhof (GGA PBE) functional. Tightly bound core electrons of Ni were represented with semicore pseudopotentials. Reciprocal-space integration over the Brillouin zone was approximated through k-point sampling with a separation of 0.4 nm^{-1} using the Monkhorst-Pack grids of 3×3×1 for both surfaces. A value of 0.08 for both charge and spin density mixing with direct inversion in the iterative subspace (DIIS) and orbital occupancy with thermal smearing of 0.002 Ha were used. The formal spin for the Ni atoms was initially set to +2.[S17] The orbital cutoff distance of 0.4 nm was set for all atoms. The Tkatchenko-Scheffler (TS) method with an sR parameter of 0.94 was used for a better description of van der Waals interactions at different surface coverages.[S15] All vibrational frequencies are reported as calculated without any adjustments, and the calculated vibrational frequencies are summarized in Table S2-S3.

For NNH_x species, we focused on NNH and NNH_2 species since these intermediates were previously identified experimentally.[S18-S19] NHNH, an isomer of NNH_2, was also studied. However, based on our DFT calculations, NNH_2 was thermodynamically more stable than NHNH on both Ni and Ni_3N surfaces by 25 and 7 kJ/mol, respectively, which indicates the surface coverage of NHNH should be very low. Therefore, the contribution of NHNH was not considered in the reaction kinetics and in the in-situ DRIFTS experiments.

2. Supplementary Results and Discussion

The formation of Ni nitride on Ni powder catalysts was identified by ex-situ Raman and TPD experiments (Figures S4 and S5). The N 1s HRXPS results show that Ni nitride was formed on Ni foil surfaces after N_2-plasma treatment. The Ni nitride structure is reported to be very sensitive to O_2, or moisture in the air, and therefore, the Ni powder catalyst was transferred after the N_2-plasma treatment to our custom-made Raman cell inside of a glove box without exposure to air. The Raman cell was kept under vacuum during the Raman spectra acquisition. A noticeable Raman band was observed at 525 cm^{-1}, which is attributed to Ni nitride (Figure S4).[S20] For the sample treated under same temperature and N_2 feed, but without turning on the plasma discharge, the Raman band at 525 cm^{-1} was not observed, indicating plasma is required under our conditions of interest to form a Ni nitride surface (Figure S4).

The formation of Ni nitride on Ni powder catalysts was further confirmed by TPD experiments (Figure S5). No N_2 desorption peak was found without N_2-plasma pretreatment. The desorption results agree with our Raman results and are as expected. Without plasma treatment, molecular N_2 only weakly physisorbs with an adsorption energy of 27-35 kJ/mol.[S21] The weakly

bound N_2 molecules desorb easily during Ar purging, and therefore, no N_2 desorption peak was expected for the Ni sample without N_2-plasma exposure. With N_2-plasma treatment, a N_2 desorption peak was observed at 630 K (Figure S5). This N_2 desorption peak is due to the thermal decomposition of Ni nitride at 630 K, which is consistent with previous studies of the decomposition temperature of Ni nitride (676 K) obtained by thermogravimetric analysis (TGA) experiments.[S22] Similar stable N-containing species were found by temperature programmed surface reaction (TPSR) experiments for SiO_2-supported Fe, Co, Ni, and Pt catalysts treated by N_2-plasma.[S23] The TPD experiments reported herein demonstrate that the Ni nitride structures are thermally stable at the temperatures utilized for reaction testing, ex-situ characterization, and the sample transfer procedures. However, Ni nitride formed can react with H_2 or H_2-derived activated species at low reaction temperatures and form NH_3.[S23-S24]

In mass spectrometry, signals at m/z = 28 could be due to CO and/or N_2, and so we monitored m/z = 12 for C and m/z = 14 for N during the TPD experiments, which are the major fragments of CO and N_2, respectively. No C peak was found at 630 K for Ni catalyst with and without N_2-plasma treatment by TPD experiments. In contrast, a N peak was found at 630 K for Ni catalysts after N_2-plasma treatment. Therefore, the TPD peak at 630 K for N_2-plasma treated Ni catalysts must be due to N_2 produced by the thermal decomposition of Ni nitride.

The thickness of the nitride layer on Ni powder catalyst was qualitatively studied using ex-situ XRD and STEM/EDS experiments (Figure S6 and S7). The elemental bulk composition of the Ni powder catalysts treated with and without N_2-plasma remained the same, and both aligned with the bulk reduced Ni reference. In XRD, since the Ni (111) peak and Ni_3N (111) peak overlap, the two peaks at 39° and 42° were used to probe the existence of bulk Ni nitride. No bulk nitride peak was found for Ni powder treated with plasma. This indicates that the bulk structure of the pure Ni powder catalyst was not affected by plasma treatment and the Ni nitride only formed as a thick surface layer on the catalyst (Figure S6). STEM/EDS was used to further characterize the Ni powder catalysts after N_2-plasma treatment. Both Ni and N signals were observed in EDS, and the N signal was mostly found at the surface of the Ni particles (Figure S7).

In summary, we confirmed that Ni nitride structures were formed on Ni powder catalyst (Figures S4-S5). Although the catalytic performance of Ni powder and Ni foil catalysts were different, the fundamental surface chemistry and surface reaction mechanisms for Ni foil and Ni powder catalysts are closely related. Therefore, characterization and analysis in the manuscript using ex-situ HRXPS on Ni foil (Figure 1) and in-situ DRIFTS on Ni powder (Figure 3) are complementary and can be combined to provide fundamental insights into Ni catalysts used for plasma-assisted catalytic ammonia synthesis.

Table S1. Effects of N_2:H_2 ratio on plasma-assisted catalytic ammonia synthesis at 300 K, 100 Torr, 20 kHz, and 18 kV.

N_2:H_2	N_2, sccm	H_2, sccm	Total NH_3 rate, μmol/s	Plasma only rate[1], μmol/s	Plasma-catalytic rate[1], μmol/s	Plasma-catalytic rate/total rate, %	N_2 conversion, %	N number density cm^{-3}	H number density cm^{-3}
∞	200	0	0	0	0	N/A	0	8.9×10^{16}	0
9:1	180	20	0.004	0.001	0.003	74	0.001	2.6×10^{16}	2.8×10^{16}
4:1	160	40	0.015	0.009	0.006	43	0.006	3.1×10^{16}	2.5×10^{16}
2:1	133	67	0.018	0.011	0.007	40	0.009	3.0×10^{16}	2.4×10^{16}
1:1	100	100	0.020	0.014	0.006	28	0.013	3.1×10^{16}	2.3×10^{16}
1:3	50	150	0.012	0.011	0.001	3	0.016	N/A[2]	1.9×10^{16}
0	0	200	0	0	0	N/A	0	0	2.1×10^{16}

[1] Defined in Section S1.3

[2] Data was not collected.

Table S2. IR vibrational frequencies (cm^{-1}) and assignments for NH_x species.

Vibrational Mode[1]	NH_3				NH_2		NH		Experimental with N_2/H_2 plasma (Figure 3)	
	Gas Experimental[2]	Gas Calculated	Adsorbed Ni	Adsorbed Ni_3N	Adsorbed Ni	Adsorbed Ni_3N	Adsorbed Ni	Adsorbed Ni_3N	Reduced	N_2 plasma
$\nu(NH_x)$	3444, 3337	3485, 3477, 3358	3429, 3427, 3274	3513, 3450, 3278	3335, 3454	3550, 3392	3354	3398	3201	3307, 3222
$\delta(NH_x)$	1627	1632, 1627	1565, 1555	1602, 1580	1443	1401	N/A	N/A	1688, 1423	1674, 1541, 1423

[1] ν -stretch, δ-deformation

[2] Vibrational levels of gas-phase NH_3 from National Institute of Standards and Technology (NIST) database

Table S3. IR vibrational frequencies (cm^{-1}) and assignments for NNH_x species.

Vibrational Mode[1]	NNH_2		NNH		Experimental with N_2/H_2 plasma (Figure 3)	
	Ni	Ni_3N	Ni	Ni_3N	Reduced	N_2 plasma
$\nu(NNH_x)$	3373, 2810	3465, 3325	3124	3133	3201	3307, 3222
$\delta(NNH_x)$	1550, 1280	1574, 1215	N/A	N/A	1209	1224
$\nu(N\text{-}N)$	1236	1104	1145	1109		

[1] ν -stretch, δ-deformation

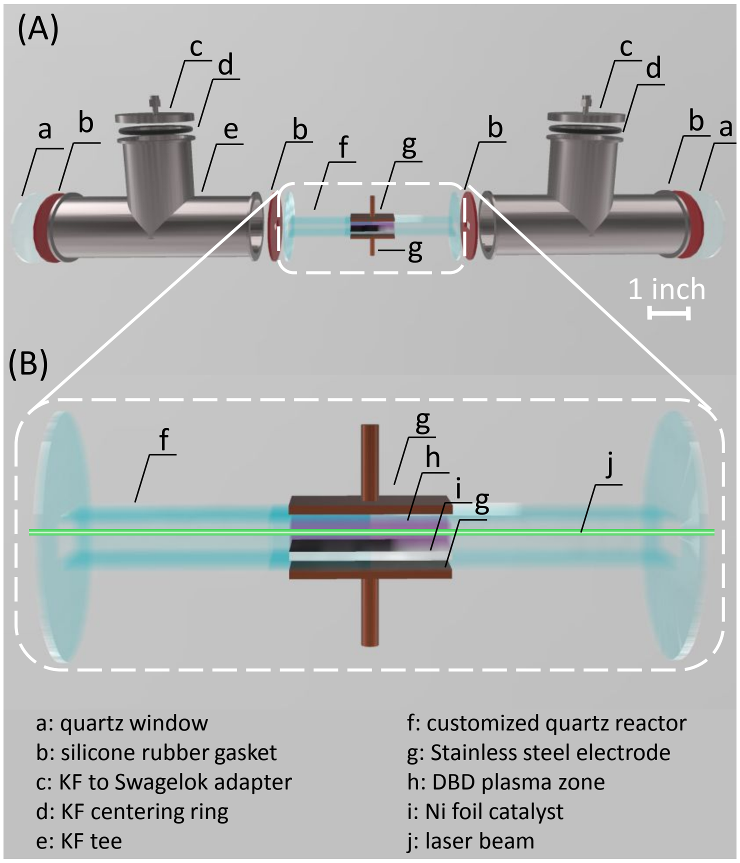

Figure S1. (A) Schematic of reactor used for ex-situ HRXPS and in-situ/operando TALIF. (B) Expanded view of the quartz reactor with 4-way optical access and the beam path for TALIF acquisition. The stainless-steel electrodes are secured and electrically isolated by adhesive-bonded ceramics (not shown). KF clamps are used to hold parts together.

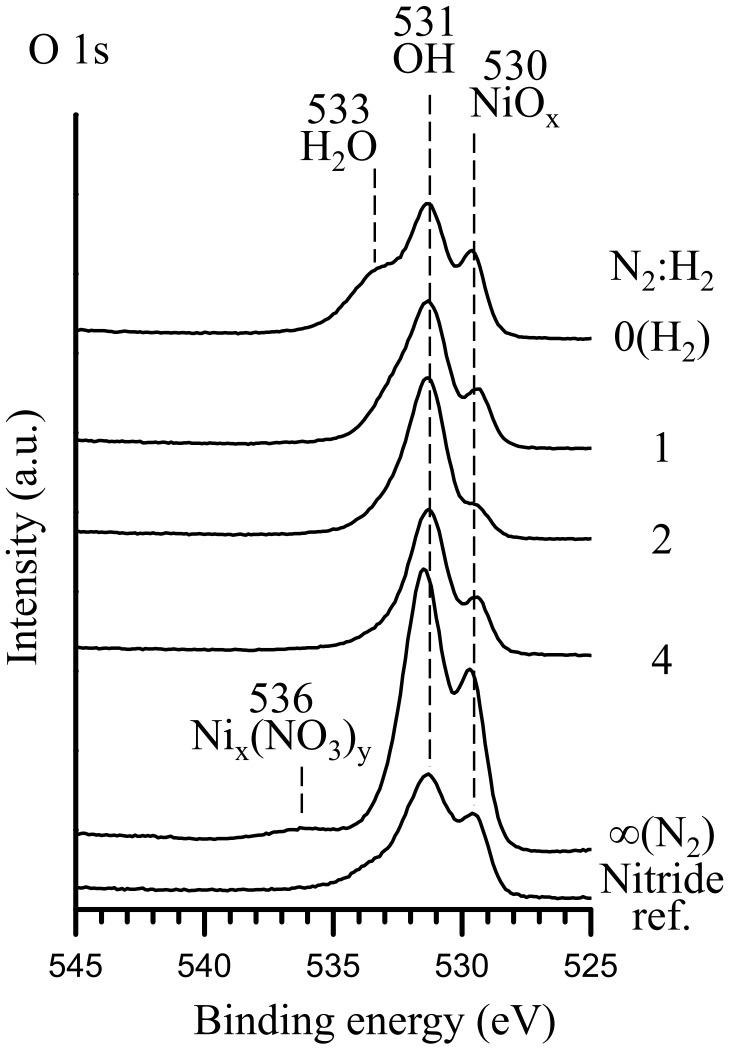

Figure S2. Ex-situ HRXPS O 1s spectra of a Ni foil catalyst after N_2-, H_2-, and N_2/H_2-plasma. Bottom spectrum is for a Ni nitride reference sample. AC DBD discharge conditions: 300 K, 100 Torr, 200 sccm, 20 kHz, and 18 kV.

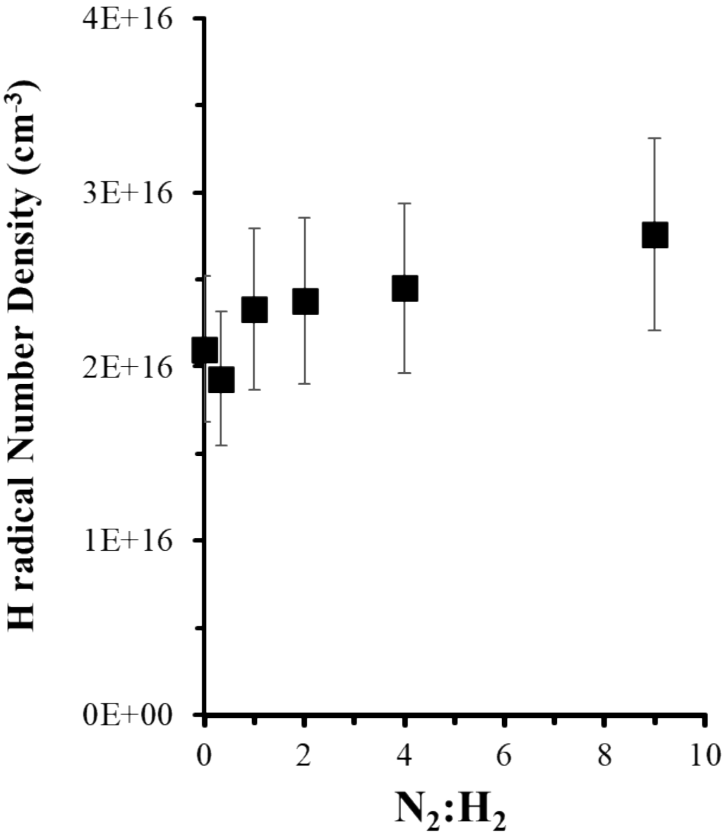

Figure S3. The dependence of the H radical number density over a Ni foil catalyst on the N_2/H_2 ratio in the DBD discharge as determined by in-situ TALIF. AC DBD discharge conditions: 300 K, 100 Torr, 200 sccm, 20 kHz, and 18 kV.

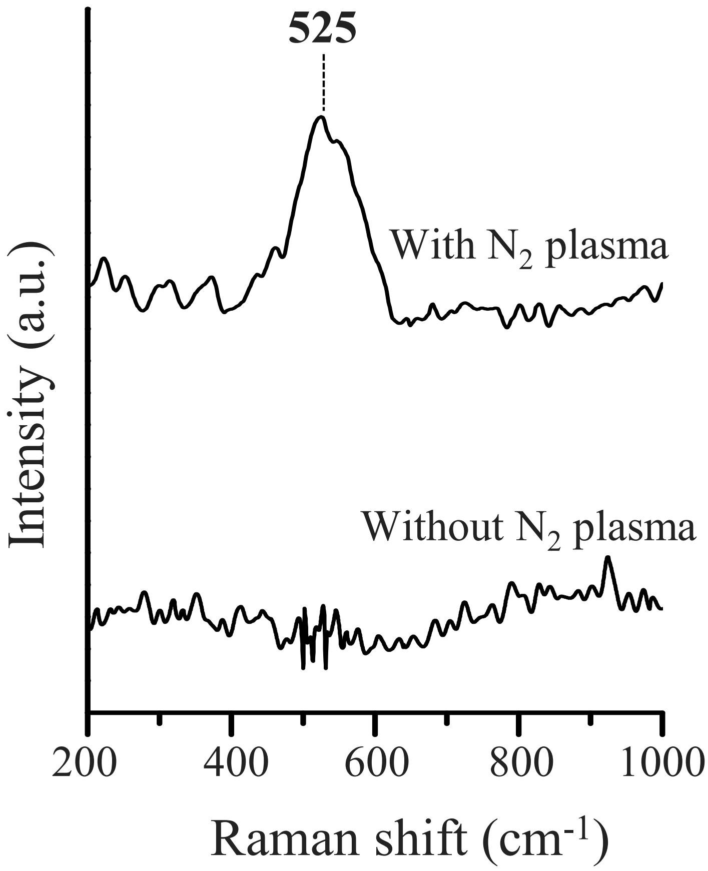

Figure S4. Ex-situ Raman spectra of pre-reduced Ni powder catalyst with and without N_2-plasma treatment. Experiment conditions: 50 sccm 40% N_2/Ar for 60 min, 100 Torr, 303 K.

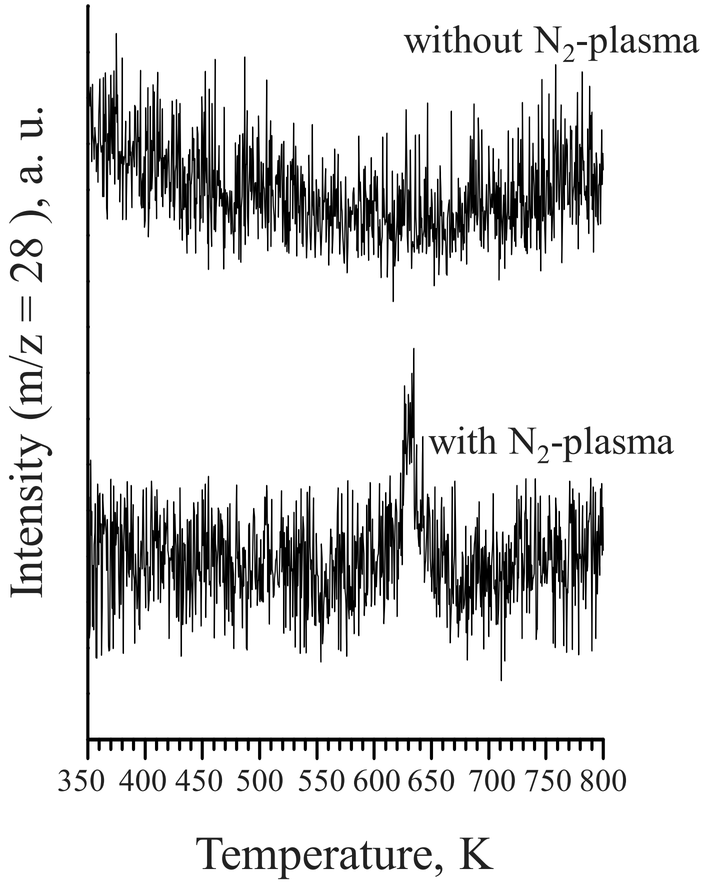

Figure S5. TPD curves from Ni powder catalyst with and without N_2-plasma treatment: 50 sccm 40% N_2/Ar for 60 min, 100 Torr, 303 K.

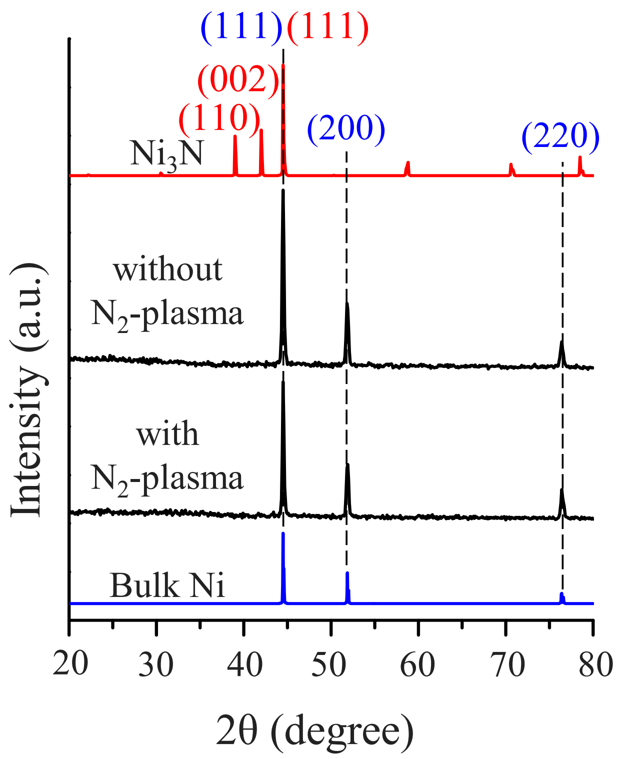

Figure S6. XRD patterns of a Ni powder catalyst with and without N_2 plasma treatment (50 sccm, 40% N_2/Ar, 60 min, 100 Torr, 303 K). The bottom pattern corresponds to a bulk Ni metal reference sample (blue), which shows three major peaks at 44° (111), 51° (200), and 76° (220). The top pattern corresponds to a bulk Ni_3N reference sample (red), which shows three major peaks at 39° (110), 42° (002), and 44° (111).

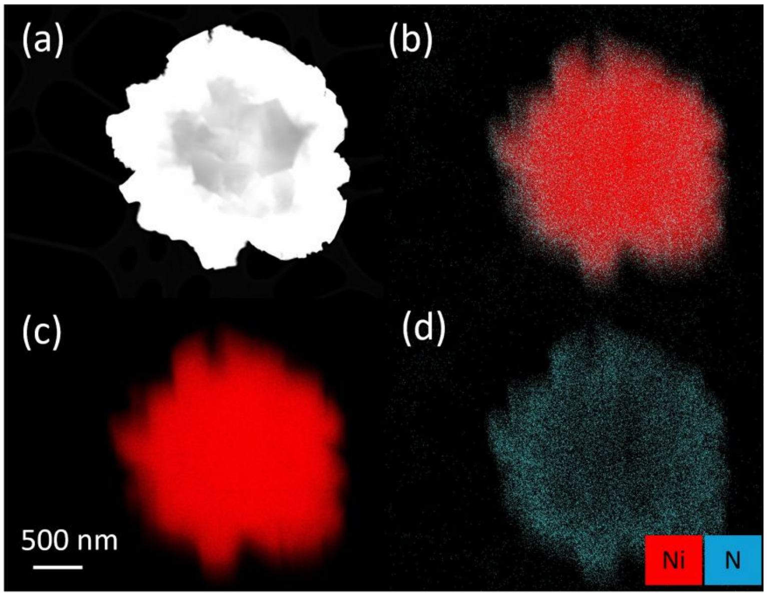

Figure S7. (a) HAADF-STEM image and (b-d) corresponding element maps for a Ni powder catalyst after N_2-plasma treatment: 50 sccm 40% N_2/Ar for 60 min, 100 Torr, 303 K.

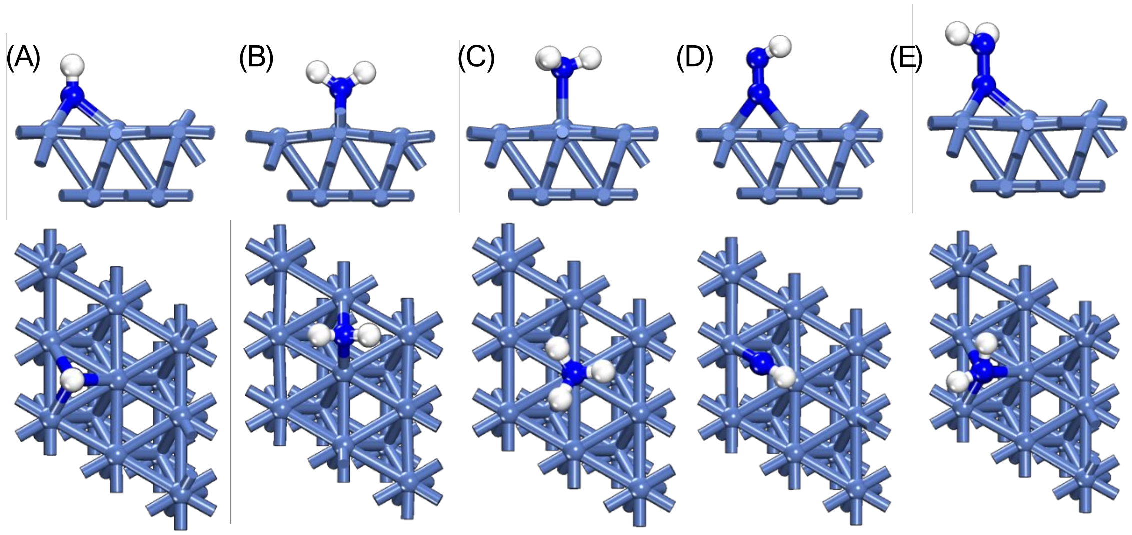

Figure S8. Optimized intermediate structures on Ni(111) obtained from DFT calculations: (A) NH adsorbed on a threefold site, (B) NH_2 adsorbed on a bridge site, (C) NH_3 adsorbed on an atop site, (D) NNH adsorbed on a bridge site, and (E) NNH_2 adsorbed on a threefold site. Side (upper row) and top (lower row) projection views are shown. Only the top two Ni layers are displayed for clarity.

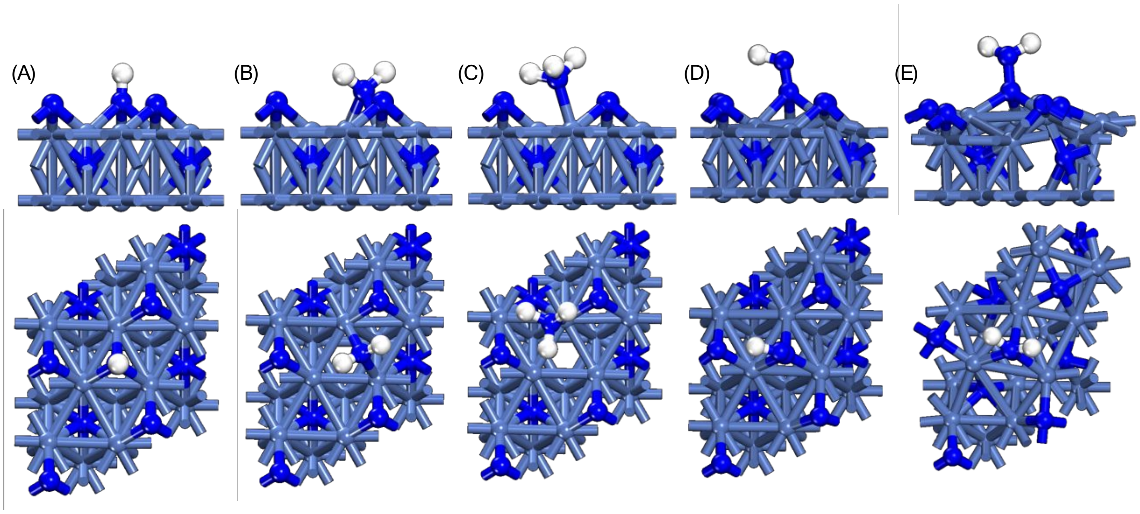

Figure S9. Optimized intermediate structures on $Ni_3N(001)$ obtained with DFT calculations: (A) NH adsorbed on a three-fold site. (B) NH_2 adsorbed on a bridge site. (C) NH_3 adsorbed on an atop site. (D) NNH adsorbed on a three-fold site. (E) NNH_2 adsorbed on a three-fold site. Side (upper row) and top (lower row) projection views. Only two top Ni nitride layers are shown for clarity.

References

S1. Liu, N.; Mao, X.; Kondratowicz, C.; Chen, T. Y.; Mei, B.; Wang, Z.; Xu, Y.; Zhong, H.; Shi, Z.; Morozov, A.; Dogariu, A.; Ju, Y., Unraveling Nonequilibrium Generation of Atomic Nitrogen and Hydrogen in Plasma-Aided Ammonia Synthesis. *ACS Energy Letters* **2024,** *9* (5), 2031-2036.

S2. Starikovskiy, A.; Dogariu, A., Calibration of Hydrogen Atoms Measurement Using Femtosecond Two-Photon Laser-Induced Fluorescence. *Opt. Express* **2025,** *33* (12), 26286-26305.

S3. Niemi, K.; Gathen, V. S.-v. d.; Döbele, H. F., Absolute Calibration of Atomic Density Measurements by Laser-Induced Fluorescence Spectroscopy with Two-Photon Excitation. *Journal of Physics D: Applied Physics* **2001,** *34* (15), 2330.

S4. Mei, B.; Wang, Z.; Thawko, A.; Liu, N.; Thompson, L.; Attinger, J.; Ju, Y., Dimethoxymethane Low- and Intermediate-Temperature Oxidation up to 100 atm. *Proceedings of the Combustion Institute* **2024,** *40* (1), 105650.

S5. Wang, Z.; Yan, C.; Mei, B.; Lin, Y.; Ju, Y., Study of Low- and Intermediate-Temperature Oxidation Kinetics of Diethyl Ether in a Supercritical Pressure Jet-Stirred Reactor. *The Journal of Physical Chemistry A* **2023,** *127* (2), 506-516.

S6. Gorky, F.; Best, A.; Jasinski, J.; Allen, B. J.; Alba-Rubio, A. C.; Carreon, M. L., Plasma Catalytic Ammonia Synthesis on Ni Nanoparticles: The Size Effect. *Journal of Catalysis* **2021,** *393*, 369-380.

S7. Barboun, P.; Mehta, P.; Herrera, F. A.; Go, D. B.; Schneider, W. F.; Hicks, J. C., Distinguishing Plasma Contributions to Catalyst Performance in Plasma-Assisted Ammonia Synthesis. *ACS Sustainable Chemistry & Engineering* **2019,** *7* (9), 8621-8630.

S8. Bayer, B. N.; Bruggeman, P. J.; Bhan, A., Species, Pathways, and Timescales for NH_3 Formation by Low-Temperature Atmospheric Pressure Plasma Catalysis. *ACS Catalysis* **2023,** *13* (4), 2619-2630.

S9. Chen, Z.; Koel, B. E.; Sundaresan, S., Plasma-Assisted Catalysis for Ammonia Synthesis in a Dielectric Barrier Discharge Reactor: Key Surface Reaction Steps and Potential Causes of Low Energy Yield. *Journal of Physics D: Applied Physics* **2022,** *55* (5), 055202.

S10. Lefferts, L., Leveraging Expertise in Thermal Catalysis to Understand Plasma Catalysis. *Angewandte Chemie International Edition* **2024,** *63* (10), e202305322.

S11. Mehta, P.; Barboun, P.; Herrera, F. A.; Kim, J.; Rumbach, P.; Go, D. B.; Hicks, J. C.; Schneider, W. F., Overcoming Ammonia Synthesis Scaling Relations with Plasma-Enabled Catalysis. *Nature Catalysis* **2018,** *1* (4), 269-275.

S12. Shao, K.; Mesbah, A., A Study on the Role of Electric Field in Low-Temperature Plasma Catalytic Ammonia Synthesis via Integrated Density Functional Theory and Microkinetic Modeling. *JACS Au* **2024,** *4* (2), 525-544.

S13. Song, F.; Li, W.; Yang, J.; Han, G.; Liao, P.; Sun, Y., Interfacing Nickel Nitride and Nickel Boosts both Electrocatalytic Hydrogen Evolution and Oxidation Reactions. *Nature Communications* **2018,** *9* (1), 4531.

S14. Zheng, Y.; Qi, Y.; Tang, Z.; Tan, J.; Koel, B. E.; Podkolzin, S. G., Spectroscopic Observation and Structure-Insensitivity of Hydroxyls on Gold. *Chemical Communications* **2022,** *58* (25), 4036-4039.

S15. Zheng, Y.; Qi, Y.; Tang, Z.; Hanke, F.; Podkolzin, S. G., Kinetics and Reaction Mechanisms of Acetic Acid Hydrodeoxygenation over Pt and Pt–Mo Catalysts. *ACS Sustainable Chemistry & Engineering* **2022,** *10* (16), 5212-5224.
S16. Trettin, J. L.; Zheng, Y.; Arumuganainar, S. E.; Caron, D. D.; Hullfish, C. W.; Koel, B. E.; Sarazen, M. L., Investigation of Pt Catalyst Dynamics under DBD Plasma Jet During CO Oxidation via Operando DRIFTS. *ACS Catalysis* **2025**, 13302-13315.
S17. Christensen, A.; Carter, E. A., Adhesion of Ultrathin ZrO_2(111) Films on Ni(111) from First Principles. *The Journal of Chemical Physics* **2001,** *114* (13), 5816-5831.
S18. Winter, L. R.; Ashford, B.; Hong, J.; Murphy, A. B.; Chen, J. G., Identifying Surface Reaction Intermediates in Plasma Catalytic Ammonia Synthesis. *ACS Catalysis* **2020,** *10* (24), 14763-14774.
S19. Zhao, H.; Song, G.; Chen, Z.; Yang, X.; Yan, C.; Abe, S.; Ju, Y.; Sundaresan, S.; Koel, B. E., In Situ Identification of NNH and N_2H_2 by Using Molecular-Beam Mass Spectrometry in Plasma-Assisted Catalysis for NH_3 Synthesis. *ACS Energy Letters* **2022,** *7* (1), 53-58.
S20. Zhang, N.; Zou, Y.; Tao, L.; Chen, W.; Zhou, L.; Liu, Z.; Zhou, B.; Huang, G.; Lin, H.; Wang, S., Electrochemical Oxidation of 5-Hydroxymethylfurfural on Nickel Nitride/Carbon Nanosheets: Reaction Pathway Determined by In Situ Sum Frequency Generation Vibrational Spectroscopy. *Angewandte Chemie International Edition* **2019,** *58* (44), 15895-15903.
S21. Grunze, M.; Driscoll, R. K.; Burland, G. N.; Cornish, J. C. L.; Pritchard, J., Molecular and Dissociative Chemisorption of N_2 on Ni(110). *Surface Science* **1979,** *89* (1), 381-390.
S22. Baiker, A.; Maciejewski, M., Formation and Thermal Stability of Copper and Nickel Nitrides. *Journal of the Chemical Society, Faraday Transactions 1: Physical Chemistry in Condensed Phases* **1984,** *80* (8), 2331-2341.
S23. Barboun, P. M.; Otor, H. O.; Ma, H.; Goswami, A.; Schneider, W. F.; Hicks, J. C., Plasma-Catalyst Reactivity Control of Surface Nitrogen Species through Plasma-Temperature-Programmed Hydrogenation to Ammonia. *ACS Sustainable Chemistry & Engineering* **2022,** *10* (48), 15741-15748.
S24. Ye, T.-N.; Park, S.-W.; Lu, Y.; Li, J.; Sasase, M.; Kitano, M.; Hosono, H., Contribution of Nitrogen Vacancies to Ammonia Synthesis over Metal Nitride Catalysts. *Journal of the American Chemical Society* **2020,** *142* (33), 14374-14383.
