# Supplementary material for: Unraveling In-Situ Formation of Surface Nickel Nitride Structures in Plasma-Assisted Catalytic Ammonia Synthesis
Source: J Phys Chem Lett. 2026 Feb 17;17(13):3933–9. doi: 10.1021/acs.jpclett.5c03923 (PMC13051430; doi:10.1021/acs.jpclett.5c03923)
Supplement: Supplementary file 2 [file jz5c03923_si_002.pdf]

jz-2025-03923q.R1

Name: Peer Review Information for "Unraveling In-situ Formation of Surface Nickel Nitride Structures in Plasma-Assisted Catalytic Ammonia Synthesis"

First Round of Reviewer Comments

Reviewer: 1

Comments to the Author

The manuscript investigates plasma-assisted catalytic ammonia synthesis using nickel foil as a catalyst, with a focus on how different N_2:H_2 feed ratios affect ammonia yield. To elucidate the relationship between gas composition and ammonia production, the plasma is characterized using laser-induced fluorescence. The results indicate that the densities of hydrogen and nitrogen radicals do not vary significantly with N_2:H_2 ratio. XPS and DRIFTS measurements performed on nickel foil and nickel powder reveal the formation of nickel nitride upon exposure to an N_2–H_2 plasma, leading the authors to propose that nitrogen vacancies act as the active sites for the plasma-catalytic reaction.

The topic is interesting, and I recommend publication after the comments below have been addressed.

Comments

1. In the Introduction, the authors state: “In addition, the presence of plasma can shift the ammonia synthesis volcano curve peak away from the benchmark Ru catalyst and toward non-noble catalysts such as Co and Ni.” This statement may be misleading. While Ru-based catalysts are highly active for ammonia synthesis, the industrial benchmark catalysts are iron-based. Please rephrase this sentence to clearly distinguish between highly active laboratory catalysts and industrial benchmark systems.

2. The variation in plasma-catalytic ammonia yield between the different N_2:H_2 ratios appears relatively small. From the data presented, I estimate a variation of approximately 15% between ratios of 1:1, 2:1, and 4:1. Could the authors comment on whether these differences are statistically significant and reproducible?

3. While reading the manuscript, I wondered about the laser beam spot size and beam geometry used for the laser-induced fluorescence measurements. Radicals located close to the catalyst surface are likely more relevant for the catalytic performance than those further away. Please specify the laser beam size in the Methods section and consider adding the laser beam path to the schematic in Figure S1 to facilitate comparison with other plasma-catalytic reactor configurations.

4. I have a comment regarding the XPS fits shown in Figure 2. In the N 1s spectrum after exposure to pure N plasma, there appears to be a clear shoulder around 400 eV. This feature is not included in the fit nor discussed in the manuscript. Could the authors comment on the possible origin of this peak?

5. Regarding the fitting of the Ni spectra, I doubt the presence of Ni^{3+} in some of the reported spectra. Satellite features associated with $Ni(OH)_2$ and Ni^{2+} are frequently misassigned as Ni^{3+} in the literature. I recommend consulting the work of Mark Biesinger, who has published extensively on the analysis and interpretation of Ni XPS spectra. I understand that this aspect is not the main focus of the study and may arise from surface oxidation or water adsorption during air exposure. However, I raise this point to help avoid potentially misleading Ni 2p peak assignments.

Author's Response to Peer Review Comments:

Response to reviewers

We thank the reviewers for their comments, which have helped us to improve the quality of our manuscript. We have replied in this document to these comments and also made appropriate changes in our revised manuscript to address these comments where needed. With these changes,

we now believe that our manuscript is suitable for publication, and we look forward to your positive response.

Reviewer#1:

The manuscript investigates plasma-assisted catalytic ammonia synthesis using nickel foil as a catalyst, with a focus on how different N_2:H_2 feed ratios affect ammonia yield. To elucidate the relationship between gas composition and ammonia production, the plasma is characterized using laser-induced fluorescence. The results indicate that the densities of hydrogen and nitrogen radicals do not vary significantly with N_2:H_2 ratio. XPS and DRIFTS measurements performed on nickel foil and nickel powder reveal the formation of nickel nitride upon exposure to an N_2–H_2 plasma, leading the authors to propose that nitrogen vacancies act as the active sites for the plasmacatalytic reaction.

The topic is interesting, and I recommend publication after the comments below have been addressed.

Authors Response:

We appreciate the positive general comments in the comments in the review of Reviewer #1, and the important specific comments included are addressed in detail below.

1. In the Introduction, the authors state: "In addition, the presence of plasma can shift the ammonia synthesis volcano curve peak away from the benchmark Ru catalyst and toward nonnoble catalysts such as Co and Ni." This statement may be misleading. While Ru-based catalysts are highly active for ammonia synthesis, the industrial benchmark catalysts are iron-based. Please rephrase this sentence to clearly distinguish between highly active laboratory catalysts and industrial benchmark systems.

Authors Response:

We agree that it is necessary to distinguish between the optimal laboratory catalysts and the industrial benchmarks. We have addressed this comment in the revised manuscript as below:

Page 3, line 13-15:

In addition, the presence of plasma can shift the ammonia synthesis volcano curve peak away from the laboratory benchmark Ru catalyst and toward non-noble catalysts such as Co and Ni.

2. The variation in plasma-catalytic ammonia yield between the different N_2:H_2 ratios appears relatively small. From the data presented, I estimate a variation of approximately 15% between ratios of 1:1, 2:1, and 4:1. Could the authors comment on whether these differences are statistically significant and reproducible?

Authors Response:

In terms of reproducibility at such low conversions, we repeated these experiments multiple times to ensure the differences were meaningful. The reported reaction rates in Figure 2A and Table S1 are the average of at least three independent runs. To demonstrate the reproducibility of catalytic testing for Ni, the reaction rates of each experiment with N_2:H_2 ratios from 4:1 to 1:1 are shown in the table below (units: µmol/s):

N_2:H_2	4:1	2:1	1:1
Run1	0.014	0.019	0.021
Run2	0.016	0.016	0.021
Run3	0.015	0.018	0.019
Run4	0.014		0.020
Run5	0.016		
Average	0.015	0.018	0.020
STDEV(1σ)	0.0011	0.0013	0.0012

Based on these results, we confirm that the trend shown in Figure 2A is reproducible. We note that reaction rates for N_2:H_2 ratios at 2:1 and 1:1 are close to each other. The difference between these two reaction rates is still larger than those of the N and H radical number densities (Figure 2B and S3). These results support our conclusion that the measured ammonia formation was not controlled by the radical number densities alone.

3. While reading the manuscript, I wondered about the laser beam spot size and beam geometry used for the laser-induced fluorescence measurements. Radicals located close to the catalyst surface are likely more relevant for the catalytic performance than those further away. Please specify the laser beam size in the Methods section and consider adding the laser beam path to the schematic in Figure S1 to facilitate comparison with other plasma-catalytic reactor configurations.

Authors Response:

We agree with the reviewer that radicals close to the catalyst surface are more relevant for catalytic performance, and a 1-D vertical scan would be beneficial to understand the concentration of N and H atoms near the catalyst surface. However, due to the limitations in our laser source and reactor design, we must position the laser in the middle of the reactor to ensure an acceptable signal-to-noise ratio. In addition, as summarized in a review article Ref. [4], it remains challenging to conduct spatial resolved laser diagnostic and to probe near-surface plasma chemistry experimentally.

Therefore, in this manuscript, we focus on probing the influence of reactants, N_2:H_2 ratios, on N and H radical number densities in the bulk plasma region and their impact on ammonia generation. We are continuing to develop a new reactor that enables in-situ spatially resolved laser

diagnostic experiments to study the radical spatial distribution for plasma-assisted catalytic ammonia synthesis.

In response to this comment, we have now added the laser path in Figure S1 and revised section S1.2 in the experimental section in the Supplementary Information, as given below:

The laser beam has a diameter of 4 mm before being focused by a 250-mm focal length lens and sent into the reactor. The laser was parked in the middle of DBD plasma reactor (about 3 mm above the catalyst, Figure S1) to excite the 1s $^2S_{1/2} \rightarrow$ 3d $^2D_{3/2,5/2}$ transition of the H radical or the $2p^3$ $^4S_{3/2} \rightarrow$ 3p $^4S_{3/2}$ transition of the N radical. Using Gaussian beam optics and a center wavelength of 206.65 nm (for N atoms), the beam diameter at the focal point was 16.4 μm.

4. I have a comment regarding the XPS fits shown in Figure 2. In the N 1s spectrum after exposure to pure N plasma, there appears to be a clear shoulder around 400 eV. This feature is not included in the fit nor discussed in the manuscript. Could the authors comment on the possible origin of this peak?

Authors Response:

We believe that Reviewer is referring to Figure 1 rather than Figure 2, since all the XPS spectra are presented in Figure 1. After refitting the N 1s spectrum for the pure N_2-plasma-treated Ni, we agree that small peaks are present around 400 eV. We have examined the N 1s spectra of all the repeat experiments conducted under pure N_2 plasma conditions and found that these shoulder peaks are reproducible, although their relative intensities varied.

These peaks are attributed to NH_x species (x = 1, 2, and 3) as discussed in the manuscript, although the samples were not exposed to H_2. The presence of NH_x species is likely attributable to the interactions between Ni nitride and water adsorbed on the reactor walls prior to the experiments or during the sample transfer from the reactor to the XPS instrument. Ni nitride can react with water, particularly in the presence of plasma, to form $Ni(OH)_x$ as well as adsorbed NH_x species, as indicated by our N 1s spectra and reported in the literature in Ref. [34].

We have updated Figure 1 using the same N 1s spectrum, but with revised peak fitting. In addition, we have revised the manuscript and included a relevant reference to discuss the possible origin of the NH_x species on Ni surface with pure N_2-plasma treatment. The revised manuscript now reads as follows.

Page 4, line 6-7:

Minor NH_x peaks at 398.8-400.8 eV are also observed after N_2 plasma treatment without H_2 exposure.

Page 4, line 15-18:

The presence of these NH_x peaks is attributed to the interactions between Ni nitride and water adsorbed on the reactor walls prior to the experiments or during the sample transfer

procedure. Ni nitride can react with water, particularly under plasma conditions, to form $Ni(OH)_x$ as well as adsorbed NH_x species.[34]

And the following reference is now cited in the revised manuscript:

34. Shalom, M.; Ressnig, D.; Yang, X.; Clavel, G.; Fellinger, T. P.; Antonietti, M., Nickel Nitride as an Efficient Electrocatalyst for Water Splitting. Journal of Materials Chemistry A 2015, 3 (15), 8171-8177.

5. Regarding the fitting of the Ni spectra, I doubt the presence of Ni^{3+} in some of the reported spectra. Satellite features associated with $Ni(OH)_2$ and Ni^{2+} are frequently misassigned as Ni^{3+} in the literature. I recommend consulting the work of Mark Biesinger, who has published extensively on the analysis and interpretation of Ni XPS spectra. I understand that this aspect is not the main focus of the study and may arise from surface oxidation or water adsorption during air exposure. However, I raise this point to help avoid potentially misleading Ni 2p peak assignments.

Authors Response:

We appreciate the reviewer for sharing these informative publications, which are helpful not only for this manuscript but also for our follow-up study. After reviewing the papers by Dr Biesinger, we agree that the broad peak at 856.1 eV is more likely due to a satellite feature of Ni^{2+} species, such as $Ni(OH)_2$. The presence of $Ni(OH)_x$ species also aligns with the findings discussed in the response to comment 4.

In response, we have changed the text in the revised manuscript as given below:

Page 3 line 34-35:

Further oxidized species due to Ni^{2+} (854.4 eV) were observed,[25-28] with corresponding satellite peaks at 856.1-860.9 eV.

The following additional references are now cited in the revised manuscript:

27. Biesinger, M. C.; Payne, B. P.; Grosvenor, A. P.; Lau, L. W. M.; Gerson, A. R.; Smart, R. S. C., Resolving Surface Chemical States in XPS Analysis of First Row Transition Metals, Oxides and Hydroxides: Cr, Mn, Fe, Co and Ni. Applied Surface Science 2011, 257 (7), 2717-2730.

28. Grosvenor, A. P.; Biesinger, M. C.; Smart, R. S. C.; McIntyre, N. S., New Interpretations of XPS Spectra of Nickel Metal and Oxides. Surface Science 2006, 600 (9), 17711779.
